# On the Performance of Variational Mode Decomposition-Based Radio Frequency Fingerprinting of Bluetooth Devices

**DOI:** 10.3390/s20061704

**Published:** 2020-03-19

**Authors:** Alghannai Aghnaiya, Yaser Dalveren, Ali Kara

**Affiliations:** 1 Department of Communications Engineering, College of Electronic Technology, Bani Walid, Libya; elganai.1962@gmail.com; 2 Department of Electronic Systems, Faculty of Information Technology and Electrical Engineering, Norwegian University of Science and Technology, 2815 Gjøvik, Norway; 3 Department of Avionics, Atilim University, Kizilcasar Mah., 06830 Incek, Golbasi, Ankara, Turkey; 4 Department of Electrical and Electronics Engineering, Atilim University, Kizilcasar Mah., 06830 Incek, Golbasi, Ankara, Turkey; ali.kara@atilim.edu.tr

**Keywords:** Bluetooth signals, feature extraction, RF fingerprinting, signal classification, emitter identification, variational mode decomposition

## Abstract

Radio frequency fingerprinting (RFF) is one of the communication network’s security techniques based on the identification of the unique features of RF transient signals. However, extracting these features could be burdensome, due to the nonstationary nature of transient signals. This may then adversely affect the accuracy of the identification of devices. Recently, it has been shown that the use of variational mode decomposition (VMD) in extracting features from Bluetooth (BT) transient signals offers an efficient way to improve the classification accuracy. To do this, VMD has been used to decompose transient signals into a series of band-limited modes, and higher order statistical (HOS) features are extracted from reconstructed transient signals. In this study, the performance bounds of VMD in RFF implementation are scrutinized. Firstly, HOS features are extracted from the band-limited modes, and then from the reconstructed transient signals directly. Performance comparison due to both HOS feature sets is presented. Moreover, the lower SNR bound within which the VMD can achieve acceptable accuracy in the classification of BT devices is determined. The approach has been tested experimentally with BT devices by employing a Linear Support Vector Machine (LSVM) classifier. According to the classification results, a higher classification performance is achieved (~4% higher) at lower SNR levels (−5–5 dB) when HOS features are extracted from band-limited modes in the implementation of VMD in RFF of BT devices.

## 1. Introduction

As a physical-layer security method, radio frequency fingerprinting (RFF) offers reasonable means to ensure protection against attacks from complex threats in wireless networks. Applications of RFF lie in safe radio communications, the Internet of Things (IoT), radar systems, military communications confrontation, and civilian radio monitoring [1,2,3]. RFF utilizes inherently unique-distinctive features, or so-called “RF fingerprints”, of physical waveforms transmitted from wireless devices to classify authorized users, and identify threats [4]. These features are extracted from the transient or steady-state regions of transmitted signals. 

Particularly, in transient signal-based RFF, it is necessary to detect transient signals to extract features for classifying wireless devices according to their model and manufacturer. However, the non-stationarity and nonlinear time series form of transient signals make difficulties in feature extraction. As it directly affects accuracy of classification, developing an efficient way is strictly required to extract the robust and subtle features of the transient signals. 

In order to extract these robust and subtle features of transient signals, some transform techniques based on Wavelet [5,6], Fourier Transform (FT) [7,8,9] and the Hilbert–Huang Transform (HHT) [10,11] have been proposed. Among these techniques, HHT provides an accurate way to extract subtle features by decomposing the transient signal, both in the time and frequency domain [12]. Basically, in the pre-processing part of HHT, the Empirical Mode Decomposition (EMD) technique is employed in order to convert a nonstationary signal to a series of intrinsic mode functions (IMFs). However, the mode mixing problem is apparent in the EMD technique. In addition to this drawback, HHT has a higher computational burden. To alleviate these drawbacks, Variational Mode Decomposition (VMD) has been proposed recently in Dragomiretskiy et al.’s study [13]. VMD is based on the simultaneous decomposition of modes non-recursively, both in the temporal and spectral domain. It is computationally simple, and does not suffer from any mode mixing problem. Its superiority over EMD has been reported in various applications, such as the monitoring of wind turbines [14], single hop and relaying scenarios [15], fluctuation analysis [16], and pulse radar fingerprint extraction [17]. On the other hand, regarding the performance of VMD with transient signals, VMD has been successfully demonstrated with Bluetooth (BT) devices [18].

### 1.1. Related Works

To extract transient fingerprints for wireless device identification, one of the existing techniques is Wavelet transform [5,6]. In Barbeau et al.’s study, amplitude–phase–frequency components have been extracted from BT transients collected from 10 different BT devices [5]. The frequency characteristics of BT signals has been obtained by applying Discrete Wavelet Transform (DWT). For classification, Hotelling’s *T*^2^ statistics have been employed. In Klein et al.’s study, Higher Order Statistical (HOS) features, such as skewness, kurtosis and variance have been extracted from instantaneous amplitude, instantaneous phase and instantaneous frequency characteristics of collected 802.11a OFDM signals from three different devices [6]. Device classification performance has been demonstrated at various SNR levels (−3–10 dB) by using extracted fingerprints and Multiple Discriminant Analysis (MDA) with Maximum Likelihood (ML) classification. 

Another transforming technique is FT, which has been utilized for obtaining transient fingerprints [7,8,9]. In Suski et al.’s study, Power Spectral Density (PSD)-based fingerprints have been extracted from 802.11a OFDM signal transients [7]. In data collection, three different devices have been used. Spectral correlation has been used for classification, and classification performance has been evaluated at different SNR levels (−10–20 dB). Danev et al.’s study has been proposed for identifying individual CC2420 radio transceivers [8]. Spectral Fisher-features have been extracted from the transient part of captured signals by using a linear transformation, which is derived from Linear Discriminant Analysis (LDA). In the experiments, signals have been collected from 50 sensor devices. To evaluate the accuracy of the system, the Equal Error Rate (EER) and Receiver Operating Characteristic (ROC) have been used. The method proposed in [9] is based on the energy spectrum of IEEE 802.11b transceivers’ transient signals, from which the spectral features have been extracted. To evaluate the classification performance at different SNR levels (0–20 dB), the fingerprints of eight different Wi-Fi transmitters have been classified by using a Probabilistic Neural Network (PNN) classifier.

Another transforming technique is HHT that has been reported in [10,11]. The specific emitter identification method presented in [10] is based on the Time–Frequency–Energy Distribution (TFED) of transient signals. In signal acquisition, eight devices from three mobile phone brands have been used. To identify the mobile phones, an SVM classifier has been employed. In [11], RFF has been implemented for BT signals collected from 20 mobile phone devices (five manufacturers with various models and serial numbers). The HHT has been applied to BT transients for producing TFED from which the features have been extracted. 

Three different classifiers (Complex Decision Tree, LSVM, LDA) have been utilized with the features extracted from the noisy transients at different SNR levels (5–25 dB). The performance of the classifiers has also been evaluated based on ROC and the area under the ROC curve (AUC). 

Recently, VMD has been used for the first time to decompose BT transient signals into a series of band-limited modes from which transient signals are reconstructed [18]. Then, higher order statistical (HOS) features, such as skewness, kurtosis and the variance of instantaneous amplitude, frequency and phase, are extracted from the reconstructed transients. The method is experimentally tested using the same dataset reported in Ali et al.’s study [11]. According to the results, the VMD-based RFF method provides a better classification performance, especially at lower SNR levels (5–10 dB), when compared to HHT. 

Overall, relevant experimental works have been listed in Table 1, where signal type, device number, feature types and classifier/decision type, along with SNR levels, are listed. When the table is examined, it can be concluded that the proposed work presents one of the highest number of devices (20 BT devices) with relatively lower SNR levels (down to −5 dB). 

### 1.2. Contributions

This article, as a follow up study of Aghnaiya et al.’s study, is devoted to scrutinizing the performance bounds of VMD in RFF implementation [18]. Firstly, HOS features are extracted both from band-limited modes and reconstructed transient signals individually. Then, the performance of both feature sets is experimentally tested using the database of BT devices reported in Ali et al.’s work [11]. Moreover, for performance bounds against noise, BT devices are identified at lower SNR levels (−5 to 5 dB) by employing a Linear Support Vector Machine (LSVM) classifier. It should be noted that relatively higher SNR values (5 to 25 dB) were already reported in Aghnaiya et al.’s work [18]. In this way, the lower SNR bounds, in which the VMD can achieve acceptable accuracy in the classification of BT devices, is explored experimentally. The classification performance results show that better performance is achieved (4% higher) when HOS features are extracted from the band-limited modes directly. In a certain sense, this can also be considered as an accuracy improvement in the implementation of VMD in the RFF of BT devices. As to the knowledge of the authors, the contributions of the study presented in this article can be summarized as follows:

(1) The effects of HOS features extracted from the band-limited modes and the reconstructed signal itself on classification accuracy in VMD-based RFF method are evaluated experimentally with BT devices for the first time.
(2) This study is the first report to analyze the lower SNR bounds in which the VMD can be effectively implemented in the classification of BT devices.

## 2. Variational Mode Decomposition (VMD)-based Radio Frequency Fingerprinting (RFF)

**Figure 1:**
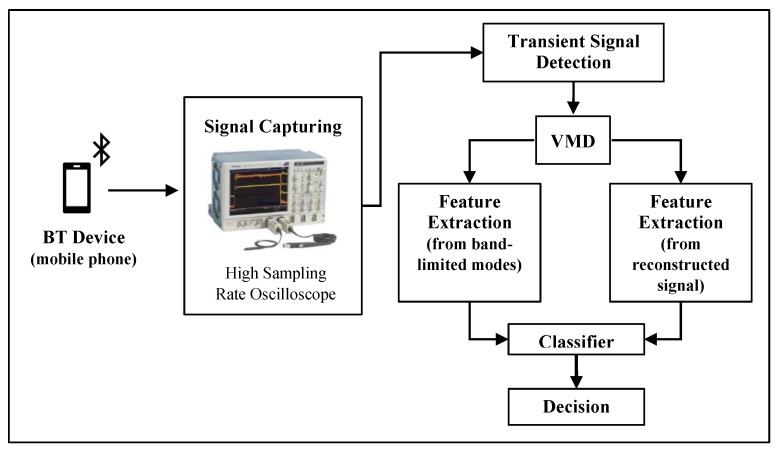
Operational diagram of the RFF implementation.

The VMD-based RFF method basically involves data collection (signal capturing) and transient detection, transient decomposition using VMD, extraction of HOS features, and classification stages. Figure 1 depicts a diagram that shows the overall process. In the following, each stage is described in the context of this study.

### 2.1. Data Collection (Signal Capturing) and Transient Detection

**Figure 2:**
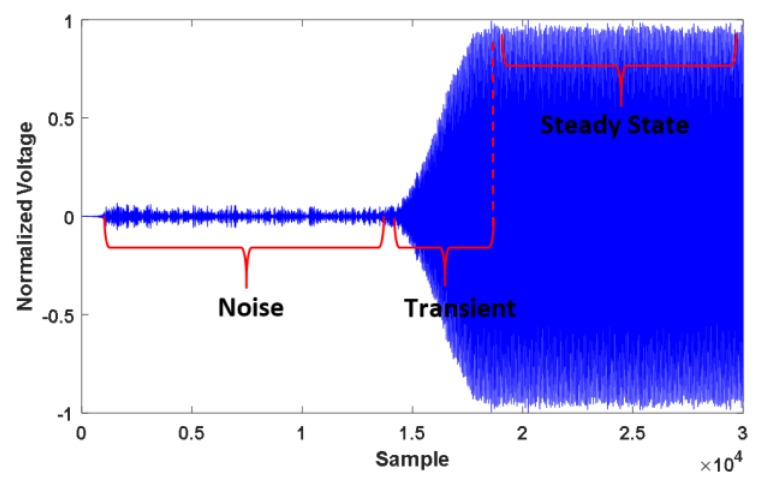
A sample recording from Bluetooth (BT) signals captured in laboratory.

In order to collect BT signals, the BT devices (mobile phones) were gathered from ten different models of five brands. Then, for each model, two different series/serial numbers were acquired. The list of devices, brands and models considered in this study can be reached from Ali et al.’s paper [11]. BT signals transmitted from these devices were captured in a laboratory environment through a high-sampling rate (20 GSPS) oscilloscope, as described in Uzundurukan et al.’s work [19]. Hence, a database consisting of 20 mobile phones with 150 transients for each device was created. Accordingly, a total of 3000 records were acquired. As illustrated in Figure 2, each of these records includes three main parts: the noisy part (channel noise), a transient signal part, and the steady state part. The recorded transients must not be required to be aligned in time (synchronization), but must have similar size as a vector for any device. The details of the process are already described in Uzundurukan et al. and Ali et al.’s works [19,20]. Here, transient detection plays an important role in RFF. This is because, if the transient signal is not fully detected, RF fingerprints will not provide the correct characteristics of the transient signal. Thus, to detect the transient properly, a band-pass filter was applied to the captured signals to remove unwanted adjacent channel signals. Then, transient detection is needed before feature extraction. In Ali et al.’s study, an efficient technique was provided for the robust transient detection of BT signals. Therefore, this technique is employed for transient detection in this study [20]. 

On the other hand, to determine SNR bounds under realistic noise conditions, where VMD can be efficiently implemented, channel noise captured in the measurements randomly added to the recorded transients at different levels. Recently, noise-related methodologies to generate additive white Gaussian noise (AWGN) noise to simulate channel noise, have been provided in Xie et al.’s studies [21,22]. However, in this study, we considered randomly varying SNR values within a range (not a single value), as this could better represent dynamic radio links of low power IoT devices, such as BT [18]. To this end, three different datasets with different SNR levels were created based on the ranges of SNR given in literature [6,7,15]: a) low SNR (−5–0 dB), b) moderate SNR (0–5 dB), and c) high SNR (5–10 dB). Note that the distribution of SNR values of each dataset (and each device) were created to follow approximately Gaussian distribution in order to prove that the performance is evaluated with varying SNR values within the range. In this way, the lower bounds of the noise performance of VMD are examined realistically with dynamic radio links for short range devices.

### 2.2. Transient Signal Decomposition

In RFF, it is possible to use HOS features directly extracted from recorded signals. This might give satisfactory results when the HOS features of both transient and steady state parts of the signal are used together, where the size of the feature set is increased greatly. However, it might not give satisfactory results when only a transient signal is used. Because these HOS features extracted from very short range, noisy transient signals (non-stationary) might not be discriminative enough. This has been studied at the very beginning, the first attempts of the work [11], based especially on the previous researches of Klein et al. and Barbeau et al [4,5]. Therefore, the VMD technique is firstly used to decompose BT transient signals (s) into a series of band-limited modes ($s_{𝓏}$, where 𝓏=1,…,Z) [13]. Mainly, this technique decomposes the given input signal into various components known as modes, which have specific properties for reproducing the input signal. It is assumed that each mode has limited bandwidth compacting around a center frequency. In the model, the bandwidth of the mode is assessed as the squared $H^{1}$ norm of its Hilbert complemented analytic signal. The analytic signal is then shifted to a baseband by complex harmonic mixing. In order to solve the variational problem, the Alternate Direction Method of Multipliers (ADMMs) approach is applied. Based on this approach, the narrow-band Wiener filtering in the Fourier domain with a filter which is tuned to the current center frequency estimate are applied to update modes iteratively. The center frequencies are then updated as the center-of-gravity of the mode’s power spectrum. Finally, the Lagrangian multiplier, which is a way of enforcing the exact reconstruction of the input signal is updated as dual ascent.

As a summary, in order to create a mode, the following scheme can be applied, as described in Dragomiretskiy et al. and Aghnaiya et al.’s works [13,18]:

- Computing the corresponding analytic signal for each mode by using the Hilbert transform
- Shifting frequency spectrum of the mode to baseband by using heterodyning
- Estimating the bandwidth of the mode by smoothing the demodulated signal, so-called Wiener filtering.

It is worth noting that the formulations and approaches provided in Dragomiretskiy et al. and Aghnaiya et al.’s works to achieve a complete algorithm are not reported here for the sake of brevity [13,18].

### 2.3. Feature Extraction

Before extracting HOS features from band-limited modes, transient signals are decomposed into three discrete band-limited modes by using VMD. Three HOS features (skewness, kurtosis and variance) are derived from signal characteristics, such as instantaneous amplitude a(n), frequency f(n) and phase ∅(n) of the band-limited modes, and then of the reconstructed signal, as in Aghnaiya et al.’s work [18]. For this purpose, an analytic signal $s^{a}\left(n\right)$ for a real-valued discrete signal in time domain s(n) can be expressed as

(1) $$s^{a}\left(n\right)=s_{I}^{a}\left(n\right)+js_{Q}^{a}\left(n\right).$$

In (1), I and Q are in-phase and quadrature components, respectively. These components are given by $s_{I}^{a}\left(n\right)=s\left(n\right),s_{Q}^{a}\left(n\right)=H\left\{s\left(n\right)\right\}$, where H{·} denotes the Hilbert Transform. Therefore, a(n), ∅(n) and f(n) can be calculated as

(2) $$a\left(n\right)=\sqrt{{\left(s_{I}^{a}\left(n\right)\right)}^{2}+{\left(s_{Q}^{a}\left(n\right)\right)}^{2}}$$

(3) $$\emptyset \left(n\right)=\tan^{-1}\left[\frac{s_{Q}^{a}\left(n\right)}{s_{I}^{a}\left(n\right)}\right]$$

(4) $$f\left(n\right)=\frac{1}{2\pi }\frac{\emptyset \left(n\right)-\emptyset \left(n-1\right)}{∆n}$$

Further, the biases superimposed by the data collection system need to be removed. Hence, the receiver-induced linear component of the instantaneous phase is eliminated, and all characteristics are normalized [11]. 

To put it simply, for each of decomposed modes, three HOS features (skewness, kurtosis and variance) are calculated from a(n), ∅(n) and f(n). In this way, nine feature vectors are created as RF fingerprints for each mode. Thus, twenty-seven feature vectors are generated from the three modes. It should also be noted that only nine feature vectors are created, as the RF fingerprint of each transient after HOS features are extracted from the reconstructed transients [18]. 

### 2.4. Classification

For each BT device, the feature vectors are divided into training and test data before classification. Here, the relationship between the feature vectors and the BT devices is established by means of the training feature vectors. The performance of the classifier, on the other hand, is estimated by using the test data. In the test data, each feature vector is supplied to the classifier without any label. Then, the label of the BT device, which is most probably be the holder of feature vector, is provided by the classifier. 

**Figure 3:**
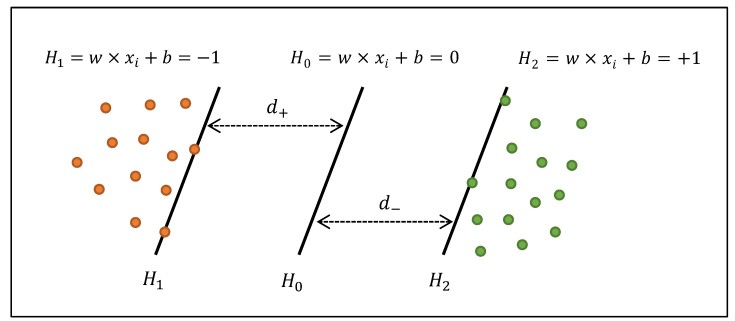
Illustration of optimal separating hyperplanes.

Similar to Aghnaiya et al.’s work, the LSVM classifier is employed for classification due to its higher performance for the BT dataset [18]. The LSVM classifier is a supervised machine learning algorithm applied for regression and classification problems. The task is to map input data (x) into high dimensional data. To achieve this task, a mapping function φ(·) and a linear function f(x)=ωφ(x)+b, where b and ω are the optimized coefficients, are used. Using f(x) enables separating data in the space, as well as generating a hyperplane [23]. In order to construct LSVM, the upper bound error is minimized when the margin between the separating hyperplanes is maximized. The separation of the data can also be achieved by utilizing the margin between these planes. Figure 3 shows the optimal separating hyperplane of the LSVM classifier. It is placed between the two separated classes (the positive, “+”, and the negative, “−“) where the margin is maximum. 

In the figure, $H_{0}$ is the median between two hyperplanes, $H_{1}$ and $H_{2}$, which are also known as the support vectors. Besides, from $H_{0}$, $d_{+}$ and $d_{-}$ are the shortest distances to the “+” and “−“ points, respectively. 

In the feature space, a decision boundary is defined by a linear discriminant function, $\hat{y}\left(t\right)$, which is expressed as

(5) $$\hat{y}\left(t\right)=\omega^{T}\varphi \left(x\right)+b.$$

In (5), ω and b are the parameters expected to be determined through a learning process of a training set, $\left[\begin{array}{ccc} \left(\begin{array}{cc} x_{1}, & y_{1} \end{array}\right), & \ldots , & \left(\begin{array}{cc} x_{n}, & y_{n} \end{array}\right) \end{array}\right]$. To define the hyperplane maximizing the margin, the following optimization problem is defined

(6) $$\min\rho \left(\omega ,\;b\right)=\frac{1}{2}\omega^{T},\;\mathrm{subject}\;\mathrm{to}\;\forall_{i}\;\;y_{i}\left[\omega^{T}\varphi \left(x\right)+b\right]$$

which can be simplified by the Lagrangian duality theory:

(7) $$\max D\left(\alpha \right)=\overset{n}{\underset{i=1}{\sum }}\alpha_{i}-\frac{1}{2}\overset{n}{\underset{i,j=1}{\sum }}y_{i}\alpha_{i}y_{j}\alpha_{j}\varphi {\left(x_{i}\right)}^{T}\varphi \left(x_{j}\right)\;\mathrm{subject}\;\mathrm{to}\forall_{i}\;\alpha_{i}\geq 0\;\mathrm{and}\;\sum_{i}y_{i}\alpha_{i}=0$$

where $\alpha_{i}^{*}$ is the solution of the given optimization problem. It provides the optimized parameters of the optimal hyperplane. The direction parameter, $\omega^{*}$ is then given as

(8) $$\omega^{*}=\sum_{i=1}^{n}\alpha_{i}^{*}y_{i}\varphi \left(x_{i}\right).$$

Accordingly, the linear discriminant function expressed in (5) can be rewritten as

(9) $$\hat{y}\left(t\right)=\overset{n}{\underset{i=1}{\sum }}y_{i}\alpha_{i}^{*}K_{x_{i},x}+b^{*}$$

where $K_{i,j}$ is Kernel function, and $b^{*}$ is the bias parameter which can be obtained from the source given in Bottou et al.’s study [24].

As mentioned in Section 2.3, the extracted feature vectors from the band-limited modes and from the reconstructed signal represent the RF fingerprints of BT devices, listed in Ali et al.’s study, each with 150 records [11]. The LSVM classifier is then trained with a training data corresponding to 40% of the total data (60 records per device). After training the LSVM classifier, it is supplied with the test data corresponding to 60% of the total data (90 records per device). The classification results are discussed in the following.

### 2.5. Results

**Figure 4:**
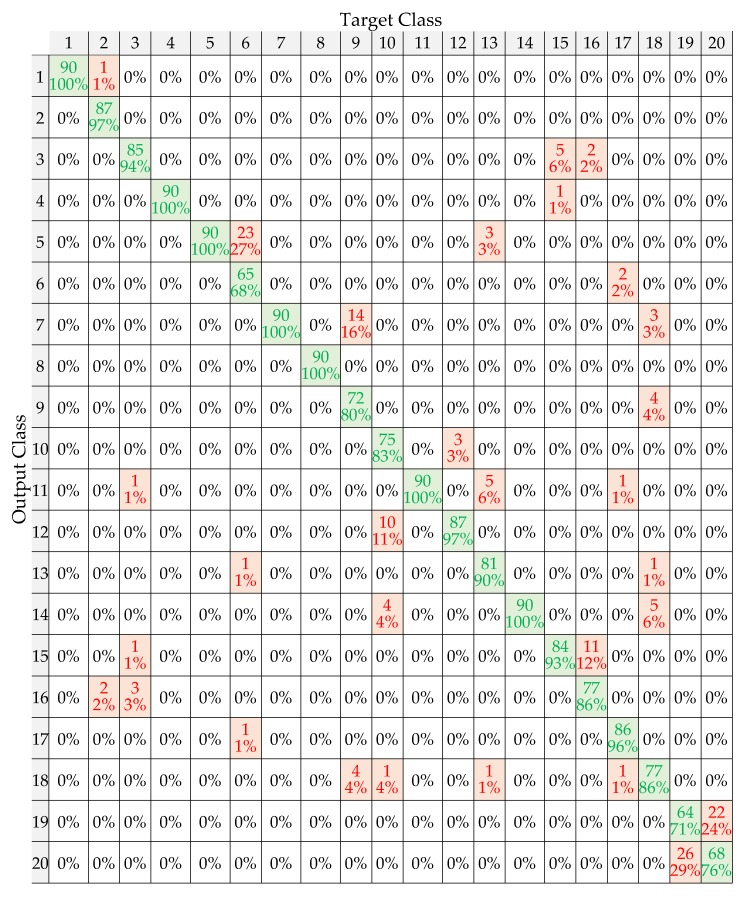
Confusion matrix (moderate SNR range).

In this subsection, the effect of extracting HOS features from the band-limited modes and from the reconstructed signal on the BT device classification performance are analyzed. To do this, the confusion matrices for 20 BT devices, which are listed in Table 1 of [11], under different SNR levels, have been generated. As an example, the confusion matrix generated for the dataset created with HOS features extracted from the band-limited modes at moderate SNR (0–5 dB) is shown in Figure 4. The diagonal elements of the matrix (green cells) represent the percentage of correct classification. In addition, the percentage of the misclassified transients of the devices are represented in red.

The overall classification accuracies at different SNR ranges (low-moderate-high) are listed in Table 2. As seen from the table, when HOS features are extracted from reconstructed transients, the classification accuracy is 67.5%, 87.3% and 96.7% at low, moderate and high SNR, respectively. On the other hand, when HOS features are extracted directly from band-limited modes, the performance increases to 70.1%, 91.0% and 97.1% at low, moderate and high SNR, respectively. These results prove that the use of HOS features extracted from band-limited modes has increased the performance of VMD-based RFF implementation, especially, at low SNR range (−5–5 dB). According to the results, it is also worth noting that VMD cannot achieve any acceptable accuracy in the classification of BT devices under the determined low SNR level (−5–0 dB).

Furthermore, when confusion matrices are examined, it has revealed that several iPhone models listed in Table I of Ali et al.’s work could be misclassified at the moderate SNR range [11]. When HOS features are extracted from reconstructed transients, the lowest classification rate of 59% was obtained. As for HOS features extracted from band-limited modes, the lowest classification rate was found to be 68%. Specifically, the highest misclassified transients were of class 6 (iPhone 6S B), which was misclassified as class 5 (iPhone 6S A). In order to analyze this result, the variance of instantaneous phase, and the skewness of instantaneous amplitude calculated for class 5 and 6, were then examined. It has been observed that these are completely overlapping when reconstructed transients are used in extracting HOS features. Contrary to this, when HOS features are extracted from band-limited modes, the variance of the instantaneous phase and the skewness of instantaneous amplitude are observed to be more separable. Therefore, it is believed that this observation could be a valid reason for achieving a higher classification performance.

## 3. Conclusions

In this study, it is aimed to scrutinize the performance bounds of VMD in RFF implementation. To this end, the effects of HOS features (variance, skewness and kurtosis of instantaneous amplitude, frequency and phase) extracted from band-limited modes, and reconstructed transient on the classification accuracy are analyzed comprehensively. While doing this, the LSVM classifier is used to identify BT devices at different SNR ranges (three ranges between −5 and 10 dB). Based on the obtained results, higher classification performance is achieved (~4% higher) at relatively lower SNR levels (−5–5 dB) when HOS features are extracted from band-limited modes. Thus, it can be concluded that the reason of achieving higher classification performance is based on the fact that band limited modes obtained from VMD decomposition possess unique characteristics of devices. Moreover, the SNR range from −5 dB to 0 dB is defined as the lower bound that the VMD can achieve acceptable accuracy in classification of BT devices. 

## Figures and Tables

**Table 1:** Summary of relevant researches of the radio frequency fingerprinting (RFF).

Technique	Ref.	Signal Type	#Device	Feature Extraction	Classifier/Process	SNR
Wavelet	[5]	BT	10	The amplitude, phase and frequency	Hotelling’s *T*^2^ statistics	*NA*
	[6]	Wi-Fi	3	HOS	MDA with ML	−3 − 10 dB
FT	[7]	Wi-Fi	3	PSD	Spectral Correlation Process	−10 − 20 dB
	[8]	Wi-Fi	50	Fisher	EER and ROC	*NA*
	[9]	Wi-Fi	8	Spectral, PCA and Amplitude	PNN	0 − 20 dB
HHT	[10]	GSM	8	TFED	SVM	*NA*
	[11]	BT	20	TFED	Complex Decision Tree, LSVM, LDA	8 − 23 dB
VMD	[18]	BT	20	HOS	LSVM	5 − 25 dB

**Table 2:** The overall classification accuracies under different SNR levels.

HOS Features	SNR Ranges		
	Low (−5–0 dB)	Moderate (0–5 dB)	High (5–10 dB)
Band-limited Modes	70.1%	91.0%	97.1%
Reconstructed Transient	67.5%	87.3%	96.7%
